# Identifying the risk of exercises, recommended by an artificial intelligence for patients with musculoskeletal disorders

**DOI:** 10.1038/s41598-024-65016-1

**Published:** 2024-06-24

**Authors:** Annika Griefahn, Christoff Zalpour, Kerstin Luedtke

**Affiliations:** 1 Department of Physiotherapy, Institute of Health Sciences, Universität zu Lübeck, Ratzeburger Allee 160, 23562 Lübeck, Germany; 2 Faculty Business Management and Social Sciences, University of Applied Science Osnabrueck, Albrechtstraße 30, 49076 Osnabrück, Germany; 3 medicalmotion GmbH, Blütenstraße 15, 80799 Munich, Germany

**Keywords:** Artificial intelligence, Risk analysis, Exercise, Musculoskeletal disorders, Health care, Medical research

## Abstract

Musculoskeletal disorders (MSDs) impact people globally, cause occupational illness and reduce productivity. Exercise therapy is the gold standard treatment for MSDs and can be provided by physiotherapists and/or also via mobile apps. Apart from the obvious differences between physiotherapists and mobile apps regarding communication, empathy and physical touch, mobile apps potentially offer less personalized exercises. The use of artificial intelligence (AI) may overcome this issue by processing different pain parameters, comorbidities and patient-specific lifestyle factors and thereby enabling individually adapted exercise therapy. The aim of this study is to investigate the risks of AI-recommended strength, mobility and release exercises for people with MSDs, using physiotherapist risk assessment and retrospective consideration of patient feedback on risk and non-risk exercises. 80 patients with various MSDs received exercise recommendations from the AI-system. Physiotherapists rated exercises as risk or non-risk, based on patient information, e.g. pain intensity (NRS), pain quality, pain location, work type. The analysis of physiotherapists’ agreement was based on the frequencies of mentioned risk, the percentage distribution and the Fleiss- or Cohens-Kappa. After completion of the exercises, the patients provided feedback for each exercise on an 11-point Likert scale., e.g. the feedback question for release exercises was “How did the stretch feel to you?” with the answer options ranging from “painful (0 points)” to “not noticeable (10 points)”. The statistical analysis was carried out separately for the three types of exercises. For this, an independent t-test was performed. 20 physiotherapists assessed 80 patient examples, receiving a total of 944 exercises. In a three-way agreement of the physiotherapists, 0.08% of the exercises were judged as having a potential risk of increasing patients' pain. The evaluation showed 90.5% agreement, that exercises had no risk. Exercises that were considered by physiotherapists to be potentially risky for patients also received lower feedback ratings from patients. For the ‘release’ exercise type, risk exercises received lower feedback, indicating that the patient felt more pain (risk: 4.65 (1.88), non-risk: 5.56 (1.88)). The study shows that AI can recommend almost risk-free exercises for patients with MSDs, which is an effective way to create individualized exercise plans without putting patients at risk for higher pain intensity or discomfort. In addition, the study shows significant agreement between physiotherapists in the risk assessment of AI-recommended exercises and highlights the importance of considering individual patient perspectives for treatment planning. The extent to which other aspects of face-to-face physiotherapy, such as communication and education, provide additional benefits beyond the individualization of exercises compared to AI and app-based exercises should be further investigated.

*Trial registration*: 30.12.2021 via OSF Registries, https://doi.org/10.17605/OSF.IO/YCNJQ.

## Introduction

According to the World Health Organization (WHO), 1.71 billion people worldwide suffer from musculoskeletal disorders (MSDs)^1^. Defined as health problems of the musculoskeletal system, MSDs are considered to be one of the world's most prevalent health problems and the leading cause of occupational illness and lost work productivity^2^. MSDs include pain and dysfunction of muscles, tendons, ligaments, cartilage, nerves and blood vessels^1,2^. They are the most common cause of chronic pain, impaired physical function and loss of quality of life^3^. The most common MSDs include low back pain (LBP), neck pain, osteoarthritis, shoulder pain, and others^3^. Various lifestyle factors (e.g. occupational exposures) and risk factors play an important role in the development of MSDs^4–6^.

Exercises are an essential component of MSD treatment^7,8^. Exercises have been shown to have several positive effects, such as pain reduction^9^, improved quality of life^10^ and well-being^11^. People in rural regions are equally affected but have limited access to adequate treatment^12,13^.One way to offer exercises for the treatment of MSD is via a mobile application that showed positive results regarding pain reduction and improved quality of life in neck pain^14,15^ and LBP^16,17^. Mobile applications can provide access to appropriate exercise for a large number of people, especially in rural areas^18–20^. However, it also has a serious disadvantage compared to physiotherapy delivered by physiotherapists: In addition to the obvious differences between physiotherapy and a mobile application, for example in terms of communication, especially for complex patients with communication difficulties or unclear diagnoses^21^. They prefer face-to-face consultations in such cases, as virtual assessments alone can lead to incomplete assessments, compromising patient safety and the effectiveness of interventions^21^. The lack of physical contact for clinical and postural assessment can lead to incomplete diagnosis and clinical reasoning^22,23^. In addition, mobile applications may have the disadvantage of providing less individualized exercises. One approach to solving the problem of less individualized exercises could be the integration of artificial intelligence (AI) that can be trained to respond to different symptom distributions, qualities and severities of MSDs. The idea of using computers to support doctors is not new and was published by Mazoué as early as 1990^24^. Positive effects of AI-based exercise therapy have already been demonstrated for neck and back pain^25,26^. Individualization of exercise programs to specific clinical presentations, as normally provided by physiotherapist and often lacking in standardized programs, can be taken into account by an AI. This includes evaluating patients’ personal needs and limitations, which may change on a daily basis. It is important that physiotherapists recommend exercises that are safe and without risk to aggravate the patient’s symptoms. This care should also be provided by the AI. In conclusion, AI offers support in the exercise management of musculoskeletal conditions when physiotherapy is not available or in-between treatment sessions. However, there is a lack of studies investigating the safety of AI provided exercise recommendations. AI provided exercises should show a comparable level of safety to physiotherapy recommended exercises. Therefore, the following research aims were derived from the challenges described above:

- To assess the risk of AI recommended exercises to increase symptoms in patients with MSDs.
- To assess the level of agreement between physiotherapists in their assessment of the risk of AI-recommended exercises.
- To analyze possible differences in patient feedback regarding exercises rated as risky or not risky to determine if these differences are significant.

## Method

### Trial design

20 physiotherapists assessed the exercises recommended by the AI to 80 (male: 32, female: 48) patient examples with MSDs. Inclusion criteria for patients were (i) musculoskeletal pain, (ii) use of the medicalmotion application between January 2020 and June 2021. There were no other inclusion or exclusion criteria for the selection of patient examples. The patient examples were provided by medicalmotion GmbH and the patients were recruited through various channels, including health insurance companies and physiotherapists. The patients exclusively received exercises via the AI and did not additionally consult physiotherapists or other health professionals. The retrospective patient examples provided with the exercises were evaluated by the physiotherapists. All users consented to the collection of data by agreeing to medicalmotion GmbH's terms of use. In addition, patients were informed that the suggested exercises were selected by an AI. This information was provided during the registration process through a welcome message explaining that the recommended exercises were generated by an AI system. A detailed description of the patient examples, their average age, well-being, and pain intensities is provided in Table 1.

**Table 1:** Characteristics of the patient examples.

Variable	Mean (SD)	Min	Max
Age (years)	51.84 (15.53)	21	79
Well-being (NRS: 0–10)	3.21 (1.73)	0	10
Pain intensity (NRS: 0–10)	4.28 (2.12)	0	8
Number of AI recommended exercises per Patient	11.8 (0.92)	6	12
Number of reported pain areas	5.38 (4.85)	1	29

*NRS* numerical rating scale. *AI *artificial intelligence. *MIN *minimum. *MAX *maximum. *SD *standard deviation.

**Figure 1:**
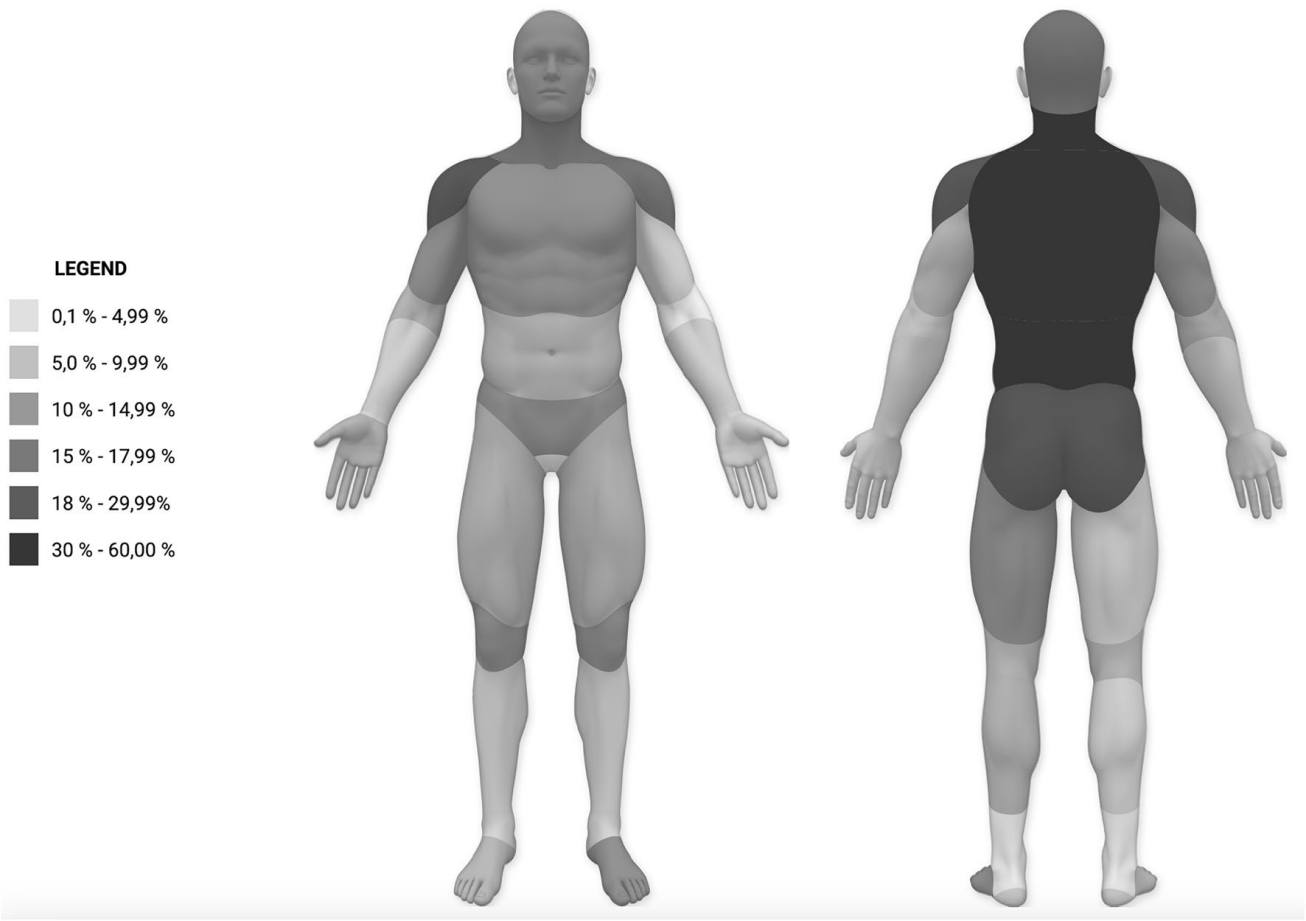
Representation of the distribution of pain areas in percent.

The most frequently reported region was the neck. The majority of patients had pain lasting > 6 months. Figure 1 shows the frequency distribution of pain areas.

In addition to the pain areas, information on current and previous diagnoses was provided. A total of 113 current diagnoses were reported. The most commonly reported diagnoses were M54.97 (dorsalgia, unspecified lumbosacral region), M54.4 (lumbago with sciatica), M53.1 (cervicobrachial syndrome), M51 (other intervertebral disc disorders) and M19.9 (arthrosis, unspecified). A total of 28 patients reported current diagnoses, of which 16 patients had at least two diagnoses. Regarding the work situation, 30 patients reported doing their job while sitting, 4 while standing, 42 while sitting and standing, and 5 reported heavy physical labor. The documented occupations included nurses (n = 10), office assistant (n = 16), mechatronics engineer (n = 15) and many more. The information on pain (quality, intensity, areas), diagnoses, information on occupation and work situation was also provided to the physiotherapists rating exercise risk as part of the questionnaire (see supplementary materials). The AI used for this project, processed all available data and included it to recommend the exercises.

For each patient example, 8–12 exercises were provided by the AI and rated by all physiotherapists. Risk in this context was defined as a potential increase in pain intensity and/or discomfort. The number of participating physiotherapists (n_Physio_ = 20) was based on the guidelines for Delphi surveys^27,28^ and the study by Esteva et al.^29^. The Delphi method is a structured communication technique developed to reach consensus among expert groups on specific topics^30^. The Delphi survey is widely used in the health care sector^31,32^. Physiotherapists were included who (i) had been working as a physiotherapist for at least 5 years, and (ii) spent at least 80% of their working time with patients with MSDs, and (iii) spoke sufficient German to answer the questionnaire.

**Figure 2:**
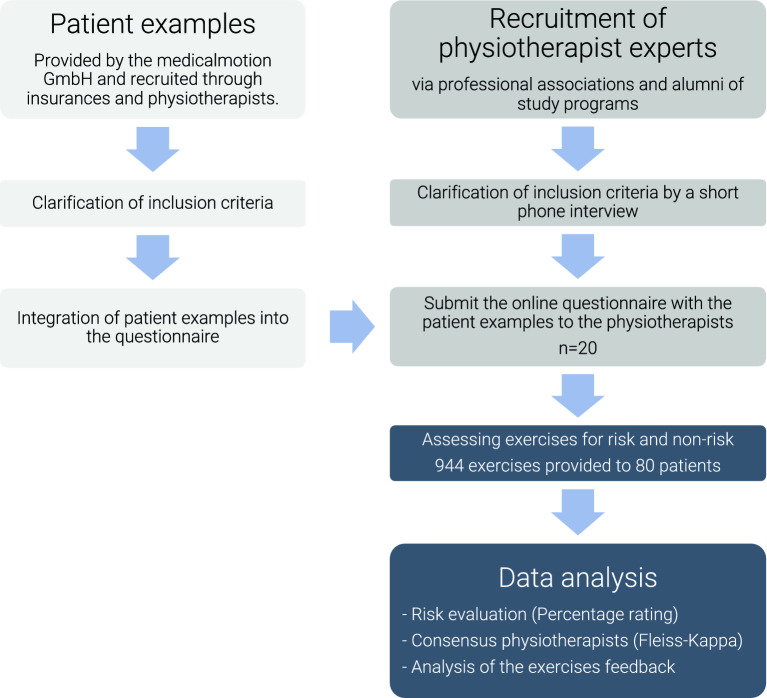
Flowchart of study procedure.

Participating physiotherapists were recruited through professional associations and graduates of the University. A telephone interview was conducted to confirm the inclusion criteria. The 80 patient examples were then made available to the physiotherapists via Limesurvey, an online survey tool, which was provided by the University of Applied Sciences Osnabrueck. Informed consent regarding data protection and data processing, as well as information about the study and the possibility of withdrawing from the study without giving any reason, was obtained before the start of the exercise evaluation. The evaluation of the patient examples was carried out anonymously, so that no conclusions could be drawn by the researchers about the identity of the physiotherapists. The data collection took place from April to December 2022. To be able to evaluate the exercises correctly, a pictorial manual was sent out. This described each exercise in detail. Figure 2 graphically depicts study procedures.

### The artificial intelligence being validated

The AI system used in this study, medico version 1.02, is a rule-based expert system with an inference engine as its central element. A rule-based expert system is a system that uses knowledge and expertise in a particular domain to make decisions or solve problems, similar to a human expert. Such a system is usually based on a set of rules provided by one or more experts in the field^33^. These rules determine how the system should respond to certain situations by using the relationships between different facts and assumptions in the system's knowledge base. In a medical context, the knowledge base typically consists of if–then rules derived from a human expert, including clinical guidelines, empirical evidence and best practices^33^. The inference engine, in turn, processes case-specific data representing a patient's clinical condition by applying the rules in the knowledge base. If the conditions of a rule are met, the system performs certain actions that may, for example, lead to additional knowledge about the patient's treatment. This additional knowledge can trigger further rules, which can also be processed. Finally, this reasoning typically leads to a final result that can be presented to the user in the form of recommendations, decisions or diagnostic advice^34^.

In the expert system ‘medico’, which is being validated here, the knowledge base consists of both heuristic and factual knowledge, as well as information about the respective exercises, such as exercise types, effects, regions used and intensity attributes. This ensures that recommendations can be based on exercises with the appropriate attributes and not on the basis that only certain exercises are recommended for a particular clinical picture. The exercises are grouped into three categories: Mobility, Strength and Release. Release exercises have the effect of loosening, relaxing and ‘de-stressing’ muscles and tissues. This improves and supports self-regulation. Mobility includes exercises that increase range of motion. This refers not only to individual joints, but also to complex movement functions. This functionally restores the body's ability to move within its normal range of motion. Strengthening exercises are exercises that strengthen muscles, tendons and supporting tissues.

The heuristic knowledge in this expert system includes the expert’s knowledge of how they would approach and treat certain conditions, taking into account the intensity of the pain, the duration of the pain, the type of work and the effects of the exercises, the area of the body on which the treatment should start and the intensity of the exercises. In this case, the ‘factual knowledge’ is based on anatomical principles and medical contraindications. In addition to expert knowledge, information from guidelines and reviews was also taken into account to create the expert system, such as the guideline “Interventions for the Management of Acute and Chronic Low Back Pain” by George et al. Among other things, this addresses which forms of exercise should be used for which type of LBP^35^.

Before the most appropriate daily exercises can be recommended, a number of steps are required. First, the information entered by the patient on pain intensity, pain characteristics, pain quality, pain duration and diagnoses, work, work stress and sporting activity is processed. Patients can adapt the information on pain, diagnosis and well-being to their current situation on a daily basis. This patient-related data therefore forms the input for the system. Previous interactions with the system and exercise feedback provide further input to the system.

Between three and five exercises are suggested each day, with an average duration of about 180 s. The exercises are always performed on both the affected and unaffected side, e.g. in the case of knee pain. The dosage is chosen individually by each patient. medicalmotion GmbH recommends a minimum of three sessions per week. The selection of exercises is done in two steps. The first step is to define the structure of the training plan and the second step is to fill the training plan structure with specific exercises. In the first step, the training plan structure is defined based on the user input processed by the knowledge base. Two exercise search strategies can be used to identify suitable exercises. The search strategy is based on the pain area (adjacent and target area) or on the pathology causing the pain. The 'target area search strategy' describes the location of the pain and the 'adjacent area search strategy' describes the regions close to the location of the pain. In the second step, when completing the training plan structure, the exercises are selected that best fit the specifications of the training plan structure, taking into account possible contraindications and the user's feedback on the exercises performed in the past, i.e. they must be of the appropriate exercise type and best meet the requirements resulting from the search strategy. For example, an initial reduction in pain may be observed after 5 training sessions, but the intensity of the pain then stagnates. In this case, it may be appropriate to switch to the ‘cause strategy’. Previous interactions with the system, such as skipping or cancelling exercises due to pain, and daily exercise feedback are incorporated into the exercise selection. Exercise feedback supports hypothesis-driven exercise selection and attempts to keep patient compliance high. For example, exercises with low feedback (0 out of 10 points) are blocked. Another example of the different use of the search strategy is the duration of pain, where the ‘target area search strategy’ is used for acute pain, for example.

This form of a rule-based expert-system enables the dual functionality of the system, which, depending on the context, works as a therapy recommendation system (TRS) for patients or as a clinical decision support system (CDSS) for therapists. The therapy recommendation system provides personalized therapy suggestions for patients, while the clinical decision support system supports therapists in their decision-making processes. In the context of the study, the system functions as a TRS. Recommended exercises can be accessed by patients on their smartphone through the medicalmotion app. The exercises were demonstrated to the patient with video support and detailed instructions for each exercise were provided via audio-guide.

### Outcome measures

The primary outcome of this study was the classification by physiotherapists of all exercises suggested by the AI as risk or non-risk. The physiotherapists assessed risk based on their individual clinical reasoning, taking into account the definition of risk used in this study, which was defined as a potential increase in pain intensity and/or discomfort. Exercises were categorized as: release, mobility and strength exercises. Each physiotherapist scored each of the 944 of exercises on a binary scale as risk or non-risk for the respective patient this exercise was recommended to. Each patient example included information on anatomical location (e.g., back, neck, knee), type (e.g., morning, always, night), duration (e.g., chronic, acute, subacute), intensity on a numeric rating scale (NRS) of 0–10, and quality (e.g., burning, aching) of the pain. Information was also collected on age, previously received medical diagnoses, well-being on a NRS ranging from 0 (= very bad) to 10 (= very good), sex and previous surgery. All information was provided by participating patients as part of the onboarding process for the medicalmotion application. The second outcome assessed the agreement between the physiotherapists regarding the risk assessment for each patient example. The third outcome of the study was the retrospective evaluation of the feedback, provided by patients after each exercise on how they perceived the exercises. Exercise feedback was given by patients on a Likert-Scale providing word descriptions for 11 categories. For example, the feedback questions for the strength exercises were “How well were you able to do the exercises?” with the answers ranging from “I couldn’t do the exercise at all (0 points)” to “no challenge (10 points)”. For the release exercises this was worded as “How did the stretch feel to you?” with the answer options ranging from “painful (0 points)” to “not noticeable (10 points)”. The equivalent for the mobility exercises was “How mobile did you feel?” with the answers ranging from “very bad due to pain (0 points)” to “very mobile (10 points)”. This was done for each type of exercise and for the entire list of AI recommended exercises (for each patient).

### Statistical analysis

For the statistical evaluation of exercise risk, the number of times an exercise was rated as a risk to a patient, was counted. In addition, the agreement between physiotherapists in the assessment of exercise risk per patient example was assessed using Fleiss-Kappa (κ_π_) for more than three raters or Cohens-Kappa (κ_d_) for two raters, and percentage agreement^36^. If there was a large deviation between the percentage agreement and the calculated kappa coefficient, the Brennan & Prediger’s (κ_q_) agreement coefficient was used^37^. In this case, a large deviation was defined as kappa values of κ  80.0%.The agreement was first evaluated for all 20 physiotherapists and all patient examples and subsequently divided between physiotherapists with 5–10 and > 10 years of work experience, the academic degree and continuing education. In addition, the agreement between the 20 physiotherapists was evaluated after the patient examples were subdivided according to the number of painful body parts (≤ 5 painful body parts, > 5 painful body parts). In the statistical analysis of patient exercise feedback, the means of the exercise feedback were compared between risk and non-risk exercises. The analysis was performed separately for the three types of exercises and for the overall feedback ratings. For this, an independent t-test was performed. The conditions of normal distribution and homogeneity of variance are first checked. The significance level was set at *p* = 0.05. Results are presented as mean values, standard deviations, *p*-value, and effect size. Effect size was assessed using Cohen's d. Interpretation of effect size was based on Cohen^38^. Statistical analysis of the data was performed using RStudio 2023 version 2023.6.1.524 with the package ‘stats’^39^.

### Ethics approval

Approval was obtained from the ethics committee of University of Applied Science Osnabrueck (ID: HSOS/2021/1/3). The procedures used in this study adhere to the tenets of the Declaration of Helsinki.

### Consent to participate

All participants provided written informed consent following a detailed explanation of the study’s purpose.

## Results

### Physiotherapy experts

The group of physiotherapists consisted of 9 female and 11 male experts. The mean age was 39.75 (8.57) years. The highest professional qualification was a diploma in 6 cases, a bachelor's degree in 6 cases, a master's degree in 6 cases and a doctorate in 2 cases. The mean professional experience was 14.65 (8.51) years. Continuing education ranged from manual therapy (MT) (n = 13), machine-assisted physiotherapy (KGG) (n = 11) to sports physiotherapy (n = 5) and orthopedic manual physiotherapy (OMPT) (n = 4).

### Exercise composition

A total of 944 exercises were rated per physiotherapist. The exercises differed in both, the type of exercise and the search strategy used by the AI. Table 2 lists the different types of exercises and shows how the system searched for matching exercises.

**Table 2:** Overview of exercises to be evaluated by exercise type and AI search strategy.

	Absolute	Search strategy		
		Adjacent	Cause	Target area
All exercises	944	225	356	363
Mobility exercises	358	57	117	184
Release exercises	574	165	235	174
Strength exercises	12	3	4	5

Target area = search strategy for the pain location, adjacent = search strategy for the nearby region of the pain location, cause = search strategy for the cause of the pain pathology.

### Risk evaluation

To assess risk, exercises suggested by the AI to each patient example were rated. The highest number of physiotherapists agreeing on an exercise as being of risk was four. This number was reached by three different exercises across the 80 patient examples. In contrast, 247 exercises across the 80 patient examples were rated as being of risk by only one physiotherapist. The agreement of (one/two/three/four) physiotherapists who rated an exercise as being of risk, is shown in Table 3

**Table 3:** Overview of the number of exercises rated as risk exercises by physiotherapy experts.

Consistent assessment per patient example of	Total number of risk ratings per exercises across the patient examples (absolute)	Total number of risk ratings per exercises across the patient examples (%)
One physiotherapist	247	1.31
Two physiotherapists	48	0.25
Three physiotherapists	14	0.08
Four physiotherapists	3	0.03

### Agreement of the physiotherapists

The total percentage agreement of how physiotherapists assessed exercise risk per patient was 90.5% with a κ_π_ = 0.017 (low agreement) and a κ_q_ = 0.634 (substantial agreement).

Taking into account the work experience of the physiotherapists (5–10 years versus > 10 years) and the number of painful body regions from the patients (≤ 5 areas versus > 5 areas), the percentage agreement ranged from 83.8 to 93.5%. An overview of the agreement levels is shown in Table 4.

**Table 4:** Physiotherapists ‘agreement on exercise risk.

	*n*_Physio_	*n*_Patient_	Total number of patient example ratings	Agreement on the non-risk exercises over the patient examples (absolute)	Agreement on the non-risk exercises over the patient examples (%)	Agreement on the risk over the patient examples (absolute)	Agreement on the risk exercises over the patient examples (%)	Kappa value	Brennan & Prediger (κ_q_)
Total sample									
Total	20	80	1600	1448	90.5%	152	9.5%	κ_π _= 0.017*	0.634**
Sample of physiotherapists separated by work experience									
5–10 years work experience	8	80	640	536	83.8%	103	16.2%	κ_π _= 0.023	0.468**
> 10 years work experience	12	80	960	897	93.5%	63	6.5%	κ_π _= 0.009	0.685**
Sample of physiotherapists separated by academic degree									
Diploma	6	80	480	407	84.8%	73	15.2%	κ_π _= − 0.11	0.614**
Bachelor’s degree	6	80	480	460	95.8%	20	4.2%	κ_π _= − 0.23	0.879**
Master’s degree	6	80	480	437	91.0%	43	9.0%	κ_π _= − 0.017	0.754**
Doctorate	2	80	160	143	89.4%	17	10.6%	κ_d_ = − 0.024	0.575**
Sample of physiotherapists separated by continuing education									
KGG	10	80	800	726	90.8%	74	9.2%	κ_π _= − 0.003	0.663**
MT	15	80	1200	1135	94.6%	65	5.4%	κ_π _= 0.005	0.796**
OMPT	4	80	320	267	83.4%	53	16.6%	κ_π _= − 0.33	0.429**
Sample of patient examples separated by pain body parts									
≤ 5 painful body parts	20	48	960	876	91.3%	84	8.7%	κ_π _= 0.019	0.685**
> 5 painful body parts	20	32	640	558	87.2%	82	12.8%	κ_π _= 0.006	0.529**

*Significant (*p* = 0.05). **highly significant (*p* = 0.001). *n*_*Physio*_ sample size of physiotherapists. *n*_*Patient*_ Sample size of the patient samples to be evaluated. *κ*_π_  Fleiss-Kappa. *κ*_d_  Cohens-Kappa. *MT*  manual therapy. *KGG*  machine-assisted physiotherapy. *OMPT* orthopedic manual physiotherapy.

On average, 72.4 (90.5%) patient examples had no exercise rated as a potential risk, whereas in 7.6 (9.5%) examples, at least one exercise was rated by physiotherapists to be a risk. The maximum number of patient examples rated by one physiotherapist with at least one exercise rated to be a risk was 34 (42.5%). The agreement per patient example showed that on average, 2 (1) physiotherapists agreed on a patient example. A maximum of 6 physiotherapists rated an exercise in a patient example as being of risk.

### Exercise feedback

Patient feedback varied between 4.65 (SD 1.88) and 8.00 (2.00) points for exercises rated as risk exercises and between 5.56 (1.88) and 7.50 (1.00) points for non-risk exercises. A detailed overview of the exercise feedback is given in Table 5. The statistical analysis of the exercise feedback showed that the exercise type ‘release’, when classified by physiotherapists as a risk exercise, received lower feedback (mean: 4.65 (1.88)) compared to the non-risk exercises (mean: 5.56 (SD 1.88); *p* = 0.001).

**Table 5:** Comparison of mean values of exercise feedback by exercise type.

Exercise type	Risk exercises assessed by physiotherapists			Non-risk exercises assessed by physiotherapists			Between group comparison (risk/non-risk exercises)	
	Frequency of evaluated feedback per exercise type (absolute)	Frequency of evaluated feedback per exercise type (%)	Mean (SD)	Frequency of evaluated feedback per exercise type (absolute)	Frequency of evaluated feedback per exercise type (%)	Mean (SD)	independent t-test	Effect size (d)
All exercises	247	26.16%	5.60 (2.31)	697	73.84%	5.99 (1.96)	*p* = 0.060	0.19
Release exercises	154	26.83%	4.65 (1.88)	420	73.17%	5.56 (1.88)	*p* = 0.001**	0.50
Mobility exercises	85	23.35%	6.93 (2.19)	279	76.65%	6.82 (1.96)	*p* = 0.717	− 0.06
Strength exercises	8	66.67%	8.00 (2.00)	4	33.33%	7.50 (1.00)	*p* = 0.677	− 0.34

**Highly significant (*p* = 0.001). The effect size was calculated using Cohen’s d with d = 0.20 (small effect), d = 0.50 (medium effect) and d = 0.80 (large effect). *SD* standard deviation. *d* effect size.

## Discussion

This study was the first to use physiotherapists’ agreement to assess whether AI can provide safe exercise recommendations for heterogeneous patient populations with MSDs. The results showed that after the assessment of 944 exercises by 20 physiotherapists, there was a three-way agreement between the physiotherapists on 14 exercises that were considered as being of risk (see Table 3). In this context, a “three-way agreement” is an agreement between three different physiotherapists. This translates to a rate of 99.92% non-risk exercises recommended to patients with MSD by an AI. On average, 90.5% of the patient examples had received non-risk exercise recommendations. The calculated Fleiss-Kappa for the agreement between the physiotherapists showed a low agreement with κ_π_ = 0.017 whereas the Brennan & Prediger coefficient showed a substantial agreement with κ_q_ = 0.634. This strong discrepancy suggests a kappa paradox^40^. The most common reason for a kappa paradox is that the prevalence of categories under consideration varies widely, leading to a bias in the kappa coefficient and not adequately reflecting the true consistency of the ratings^40^. In contrast, the Brennan & Prediger coefficient considers the prevalence of the categories and provides a better estimate of the true consistency of the ratings, especially when the prevalence is low. As a result, the Brennan & Prediger coefficient may provide a more accurate estimate of observer agreement than the conventional kappa coefficient^37^.

In 34 of the 80 patient examples, at least one exercise was rated by at least one physiotherapist as being a risk to the specific patient example. However, when looking at individual patient examples, on average only approximately 2 physiotherapists agreed on exercise risk. This low agreement may be explained with the varied educational background of individual physiotherapists. There is a wide range of treatment options for MSDs such as LBP^41^. The WCPT describes various ethical criteria for physical therapy in its Policy Statement on Ethical responsibilities of physiotherapists and WCPT members^42^. In the present study, although a physiotherapy expert group was used, individual backgrounds and continuing education play a critical role in the expression of core competencies to assess exercise recommendations^42^.

Statistical analysis of the retrospective exercise feedback showed that there was no significant difference between non-risk and risk exercises (*p* = 0.06) (see Table 5). For exercises classified as ‘release’, there was a significant difference between non-risk and risk exercises with *p* = 0.001 and a mean effect of d = 0.50. The results suggest that the subjective perspective of the patient, when using AI to recommend exercises, should also be considered in the treatment of MSDs. This could help to promote the therapeutic alliance. It has also been shown that the therapeutic alliance can have a positive influence on the improvement of chronic pain in face-to-face physiotherapy^43^. Taking into account the psychological aspects, another important point is the interpretation of user feedback and the reaction of the AI. If a user states that they were unable to do something, this could be for a variety of reasons ranging from psychological factors to physical limitations. If a user states, “I couldn’t do this at all”, this could indicate that the user was not in the right mood or mental state to perform the exercise, even if they were physically able to. When the AI receives this type of feedback, it is important that it takes into account not only the physical aspects but also the mental aspects. However, it is also important that the AI does not jump to the conclusion that the user was unable to perform the exercise for psychological reasons without having more information. Future versions of the AI could ask additional questions to better understand the reasons for the user's feedback before responding. Overall, an AI should try to develop a holistic and individualized approach to user feedback that takes into account both physical and psychological aspects to ensure that the exercises are effective and suitable for the user.

A scoping review by Damoiseaux-Volman et al. found that CDSS can improve patient safety, particularly in medication management, by improving both the accuracy of medication use and the appropriateness of clinician medication choices^44^. Similarly, the results of the present study suggest that the CDSS used here has little to no risk of increasing pain when recommending exercise therapy to patients with MSDs. Furthermore, a review by Sutton et al. highlighted that one of the key advantages of CDSS is their ability to adapt directly to a patient’s specific situation^45^. This adaptability is critical in the management of MSDs, ensuring that exercise recommendations are tailored to the individual needs of the patient, thereby minimizing risk and increasing the effectiveness of therapy. This also plays a major role of CDSS risk assessment in exercise recommendations for people with MSDs, as the pain situation is highly variable, especially in chronic pain patients, and the system should adapt to this^46^.

Ethical concerns are often raised about the use of AI. In the context of this study, it was shown that 99.92% of the exercises were considered safe by the physiotherapists. Conversely, this means that the use of AI to provide exercise advice can initially be considered safe, which means that a large number of people with MSDs can be reached. Despite the promising results of this study, which suggest that AI is able to provide safe exercise recommendations for patients with MSDs, it is important not to underestimate the role of the physiotherapist. It must be emphasized that the role of the physiotherapist remains crucial, especially in complex individual situations, particularly in the context of the bio-psycho-social model and in order to ensure holistic care^47^. AI can serve as a supportive tool, but does not replace the expertise and empathy of a qualified therapist who is able to take into account the specific needs and context of the patient. It is therefore necessary to consider AI as a complement to physiotherapeutic expertise in order to ensure the best possible patient care^48^.

## Limitations

Firstly, the sample size was based on recommendations for a Delphi survey, generally used in the medical field to reach consensus. In order to meet the requirements of a Delphi survey, a second or, if necessary, a third expert survey would have been necessary. In order to keep the dropout rate as low as possible, only one round was conducted using a large number of patient examples. A further validation step is required to consolidate the expert consensus. Secondly, the lack of a definition of risk is another limitation. In this study, the term 'risk' needed to be defined. Risk is defined here as a potential increase in pain intensity and/or discomfort for the individual patient for whom the exercise was recommended. This narrow definition may affect the transferability of the results to other trials, as different trials may use different risk criteria or definitions. Thirdly, in validations in other medical fields, ‘truth’ can be assumed, e.g. whether a diagnosis exists or not. In this study, the physiotherapists' expertise was used to establish a ‘truth’ for the assessment of whether an exercise presents a risk to patients or not. Fourthly, the sample had an unbalanced distribution of occupations, which could lead to bias. Nevertheless, this distribution reflects the actual distribution of occupations among employees in Germany^49^. However, it should be noted that the sample may not be representative of all occupational groups and the generalizability of the results may therefore be limited. Fifthly, the physiotherapists could only analyze the data retrospectively and there was no direct contact between the physiotherapists and the patients. Therefore, the physiotherapists had to rely on the information provided. In a future study, direct contact between physiotherapists and patients could provide further evidence of possible risks when recommending exercise.

Finally, the data from this current study cannot prove the clinical efficacy of the exercises recommended by AI, as it focused exclusively on risk assessment.

## Conclusion

The ability of AI to distribute risk-free exercises to heterogeneous patients with MSDs enables new forms of patient care and management. The increasing shortage of specialists, for example in rural areas, could be counteracted by providing early treatment for pain and MSDs until physiotherapy can be provided.

The above research objectives can be answered based on the study conducted. AI was shown to recommend exercises with no identifiable risk to patients with musculoskeletal conditions in over 99% of patient examples. This suggests that AI-based recommendations could be an alternative of creating exercise plans for individual patients without increasing pain intensity or discomfort. In addition, the study shows substantial agreement between physiotherapists in their risk assessment of AI-recommended exercises. This agreement increases confidence in the reliability of AI-based recommendations in physiotherapy practice. Finally, significant differences in patient feedback were only found for the exercise type ‘release’. This suggests that patients have different experiences and perceptions of exercises with and without risk. Understanding these differences may help to develop personalized treatment plans that take into account not only the physiological aspects but also the subjective responses of patients.

Once the risk assessment was complete, three completely new rules were derived and implemented in the AI-system, and several existing rules were adjusted in terms of parameter values. These rules concerned, among other things, the risk level of individual exercises for individual patient groups, the integration of specific blocking rules when specific pain qualities were indicated, and the recommendation of specific forms of exercise when specific pain qualities were indicated.

### Supplementary Information

Supplementary Information.

## Data Availability

The data can be requested from the corresponding author.
