# Supplementary material for: Identifying the risk of exercises, recommended by an artificial intelligence for patients with musculoskeletal disorders
Source: Sci Rep. 2024 Jun 24;14:14472. doi: 10.1038/s41598-024-65016-1 (PMC11196744; doi:10.1038/s41598-024-65016-1)
Supplement: Supplementary file 1 — Supplementary Information. [file 41598_2024_65016_MOESM1_ESM.docx]

# Supplementary Files

Example of risk assessment questionnaire for physiotherapists


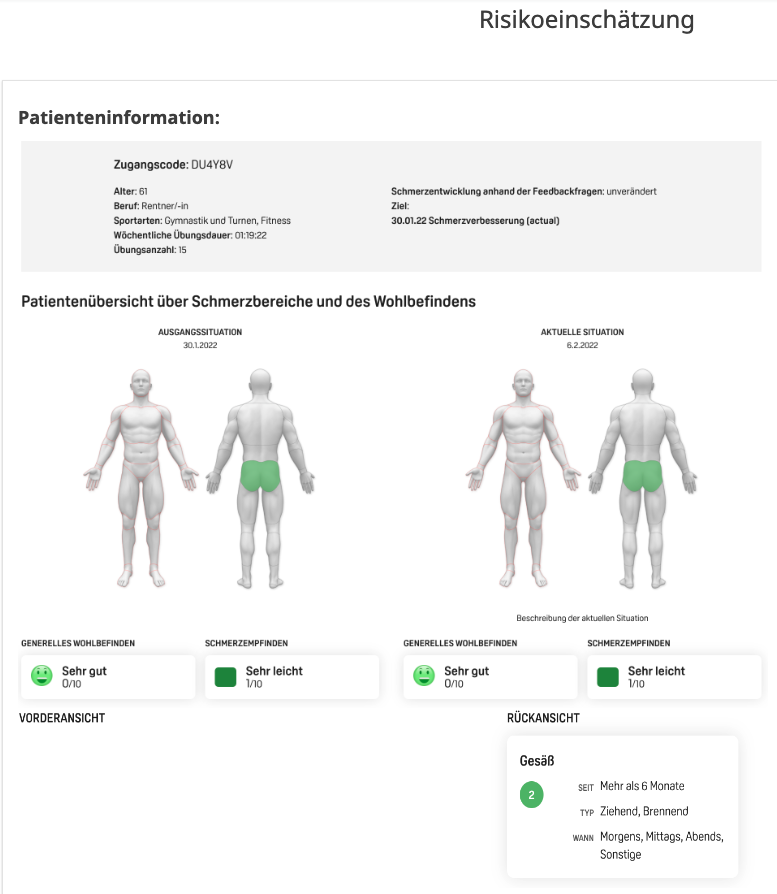


**Age:** 61

**Occupation:** Office administration

**Work situation:** sitting and standing

**Sport type:** Fitness

**Weekly exercise duration:** 01:19:22

**Exercise number:** 15

Since: more than 6 months

Type: pulling, burning

When: morning, midnoon, evening, others

Buttocks

Back view

Front view

Very light

1/10

Very light

1/10

Very good

0/10

Very good

0/10

Pain intensity

Pain intensity

General well-being

General well-being

Actual situation

02/06/2022

Initial situation

01/30/2022

**Patient overview of pain areas and well-being**

**Patient information:**


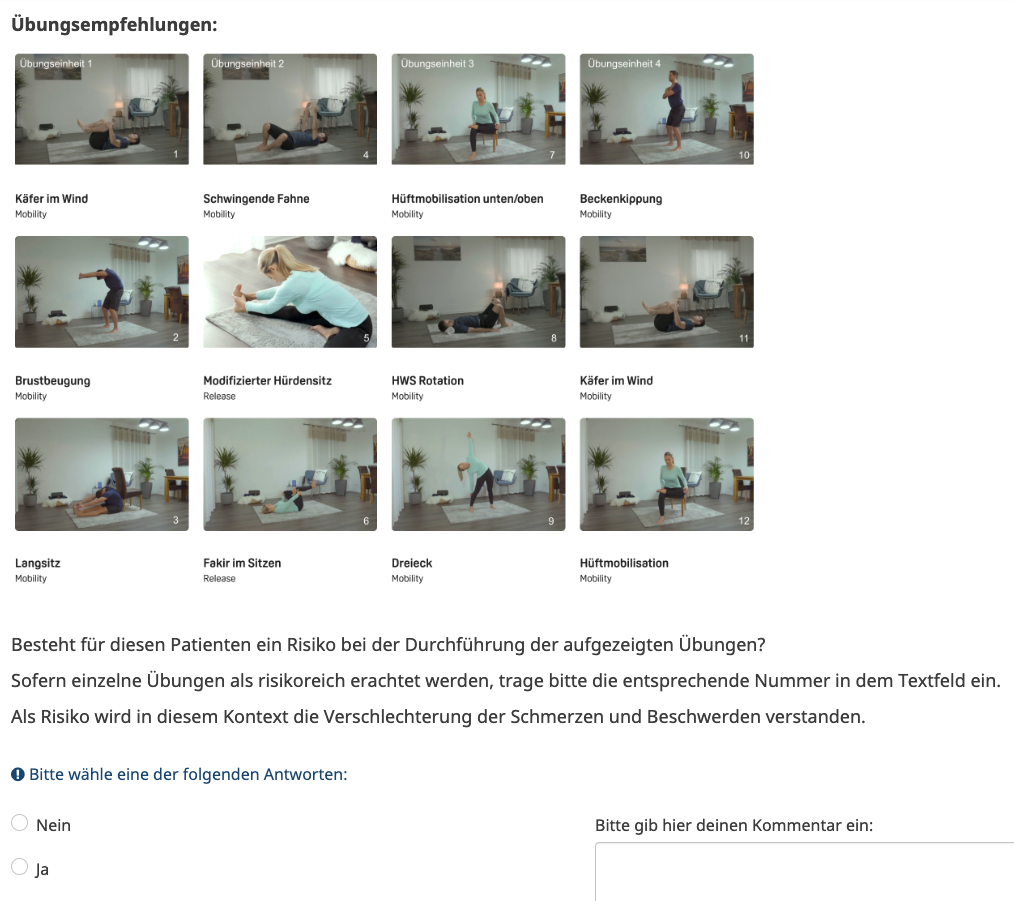


Hip mobilization

mobility

Triangle

mobility

Lying hip mobilization

mobility

Long seat

mobility

Beetle in the wind

mobility

CS rotation

mobility

Modified hurdle seat

mobility

Breast flexion

mobility

Pelvic tilt

mobility

Hip mobilization up / down

mobility

Waving flag

mobility

Beetle in the wind

mobility

Exercise Unit 4:

Exercise Unit 3:

Exercise Unit 2:

Exercise Unit 1:

**Exercise recommendations:**

Please enter your comment here:

Yes

No

Please choose one of the following answers:

Is her a risk for this patient in performing the listed exercises?

If any of the exercises are considered risky, please enter the corresponding number in the text box.

Risk in this context was defined as a potential increase in pain intensity and/or discomfort for the individual patient for whom the exercise was recommended.

© medicalmotion GmbH

Questionnaire on the medical history of the patient during the boarding process

| 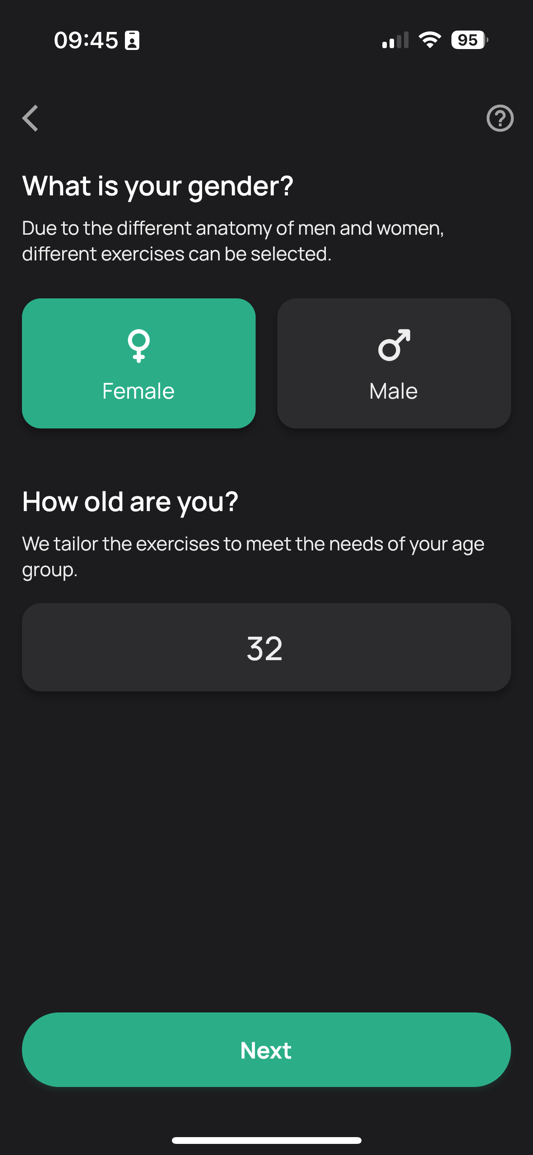 | 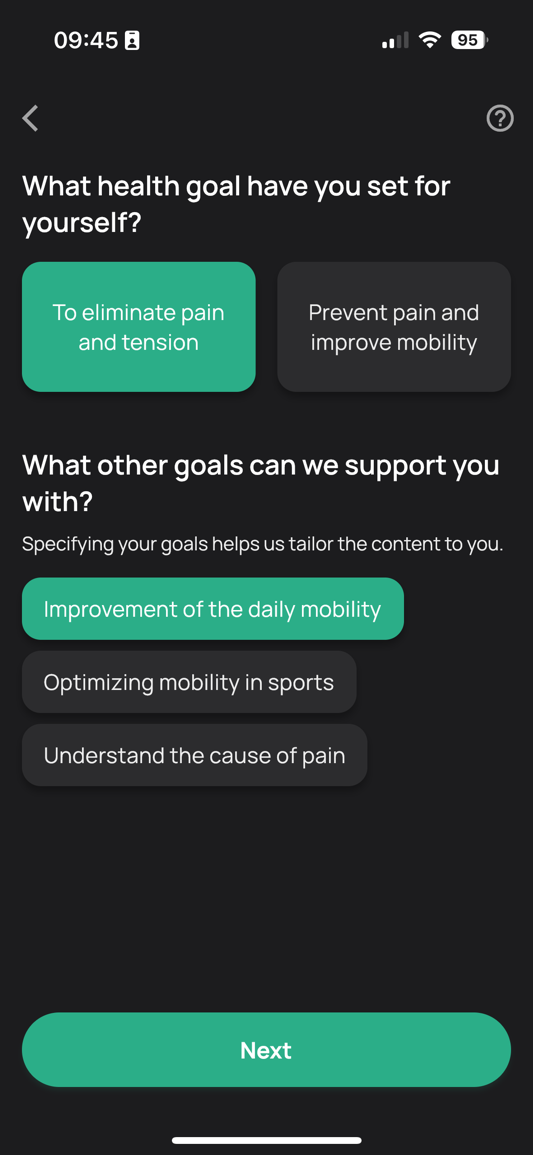 |
| --- | --- |
| 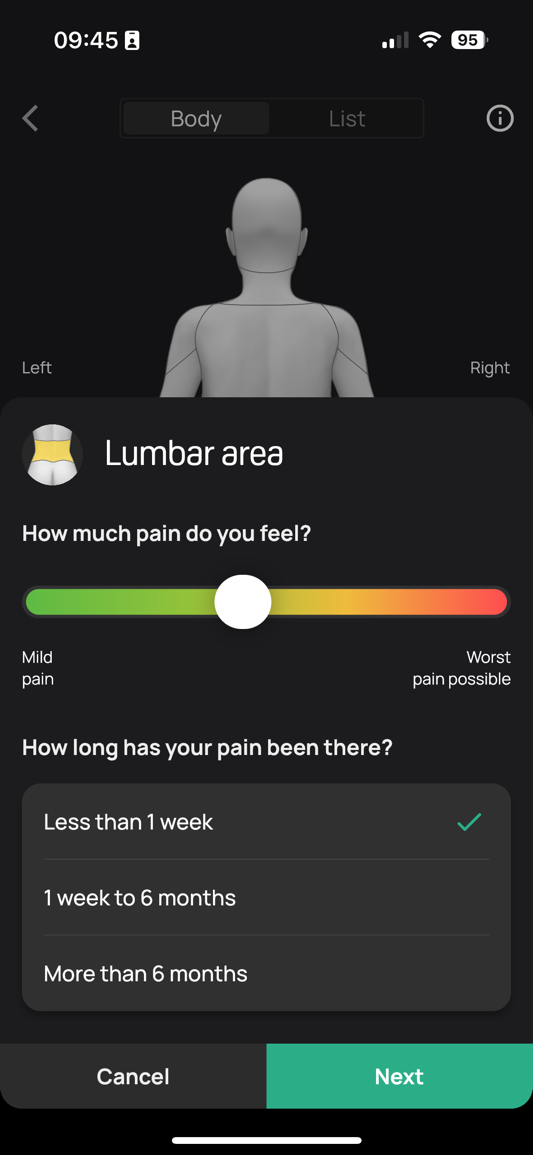 | 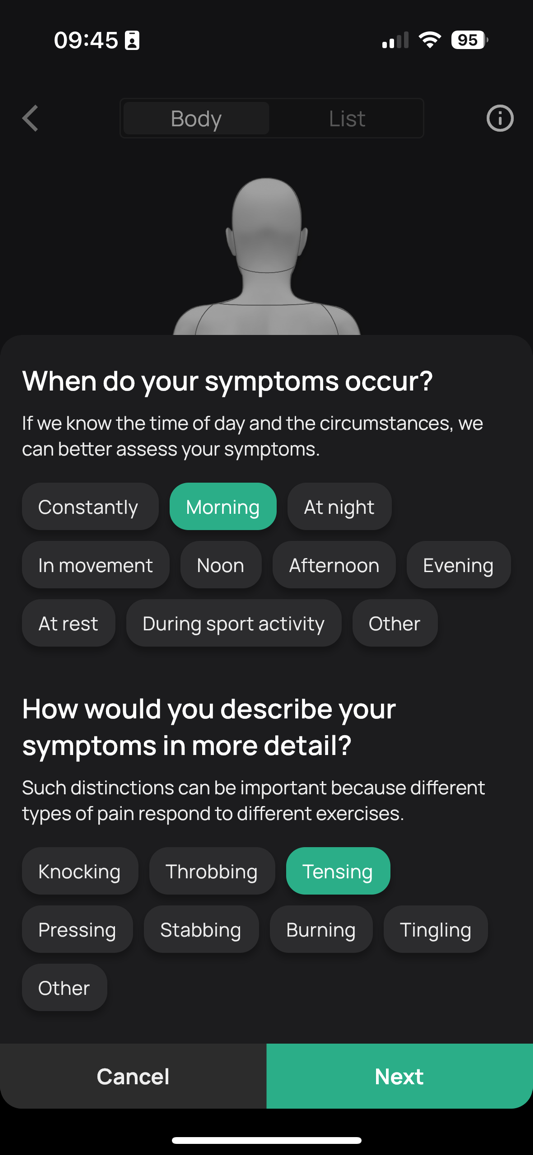 |
| 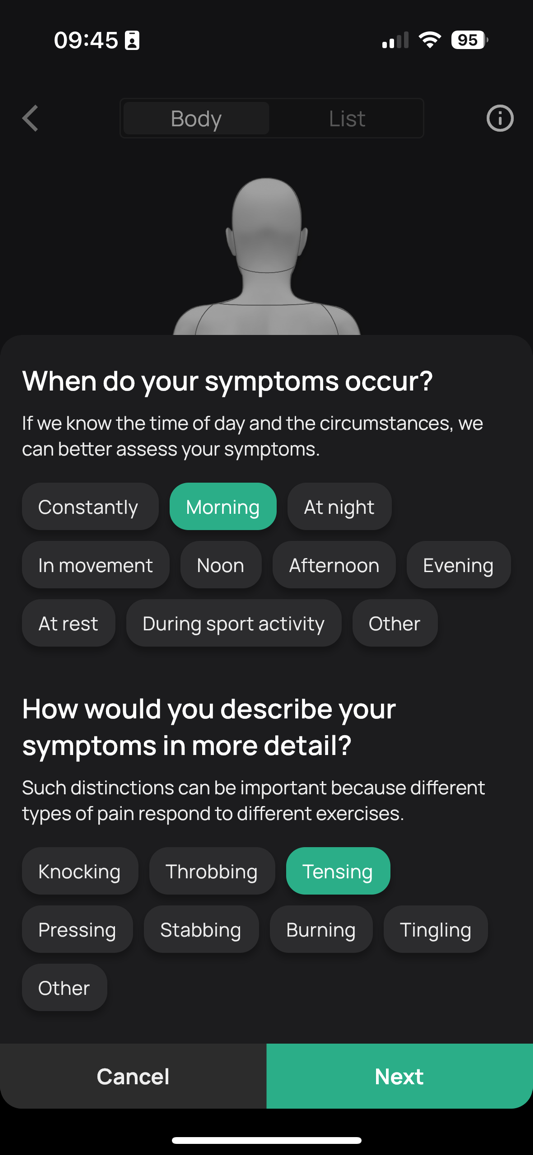 | 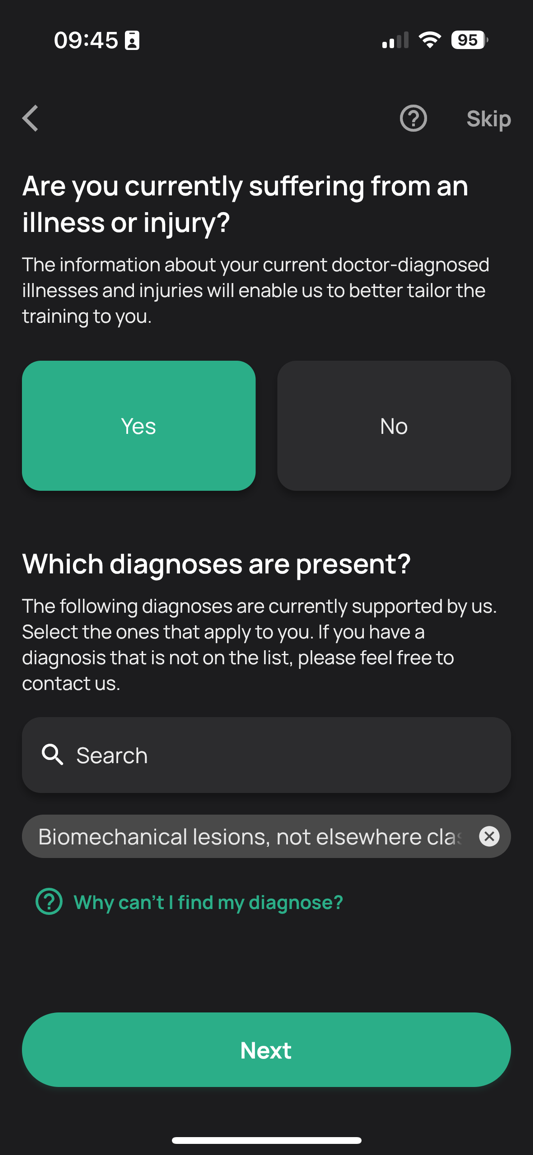 |
| 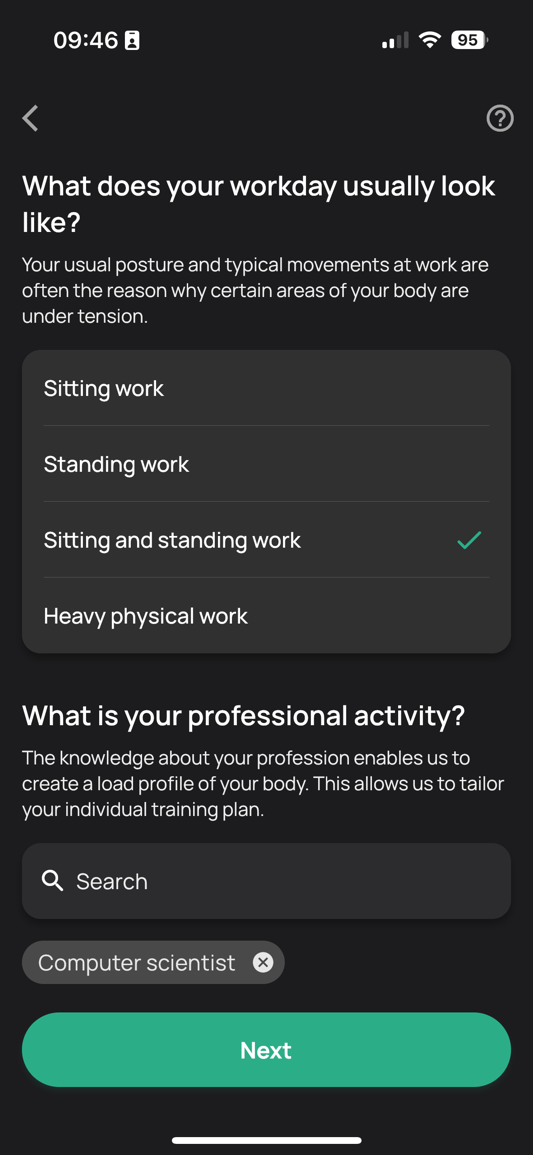 | 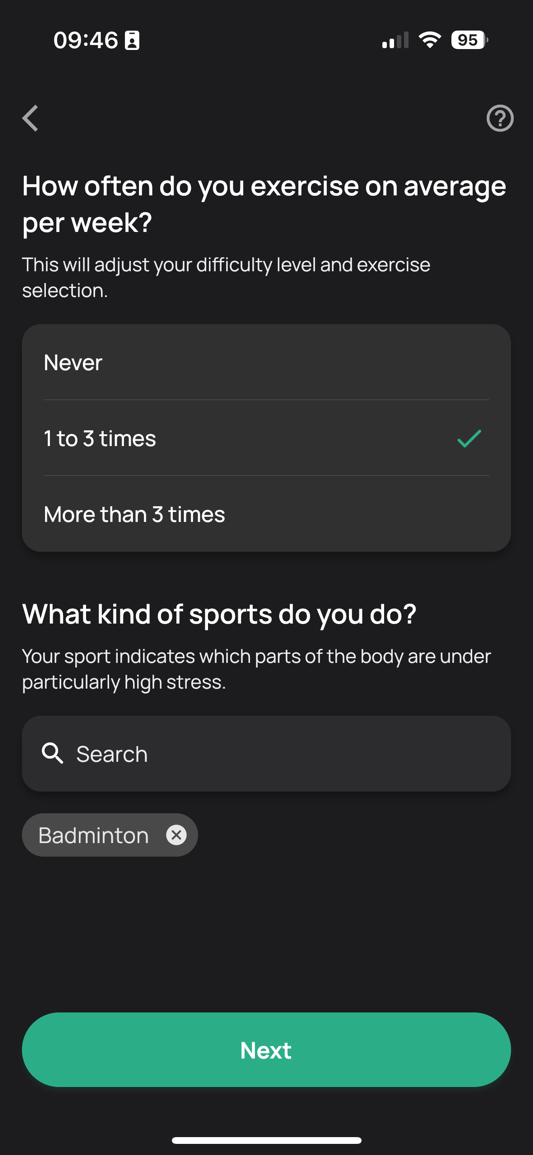 |
| © medicalmotion GmbH, Screenshots from 07.11.2023 | |
